# Supplementary material for: Low Self-Esteem and Life Satisfaction as a Significant Risk Factor for Eating Disorders among Adolescents
Source: Nutrients. 2023 Mar 25;15(7):1603. doi: 10.3390/nu15071603 (PMC10096620; doi:10.3390/nu15071603)
Supplement: Supplementary file 1 [file nutrients-15-01603-s001.zip › Supplementary Table S1.pdf]

Supplementary Table S1. The influence of age on losing more than 10 kg weight.

**Losing 10 kg or more**	**Basic descriptive statistics**							
	n	Medium	Median	Min.	Max.	Quartile I	Quartile III	Standard deviation
**Yes**	53	14,87	14,00	12,00	19,00	13,00	17,00	2,45
**No**	180	15,64	16,00	12,00	19,00	13,00	17,50	2,27
**Total**	233	15,47	16,00	12,00	19,00	13,00	17,00	2,33
	Z=-2,06 p=0,039							

Z- Mann-Whitney U test result; red color indicate significant values ($p < 0.05$)
