# Supplementary material for: Low Self-Esteem and Life Satisfaction as a Significant Risk Factor for Eating Disorders among Adolescents
Source: Nutrients. 2023 Mar 25;15(7):1603. doi: 10.3390/nu15071603 (PMC10096620; doi:10.3390/nu15071603)
Supplement: Supplementary file 1 [file nutrients-15-01603-s001.zip › Supplementary Table S2.pdf]

Supplementary Table S2. Weight loss of 10 kg or more and self-esteem

**Losing 10 kg or more**	**Basic descriptive statistics**							
	n	Medium	Median	Min.	Max.	Quartile I	Quartile III	Standard deviation
**Yes**	53	25,02	26,00	15,00	32,00	22,00	28,00	3,92
**No**	180	23,53	24,00	13,00	33,00	21,00	26,00	3,82
**Total**	233	23,87	24,00	13,00	33,00	22,00	27,00	3,89
	Z=2,55 p=0,010							

Z- Mann-Whitney U test result; red color indicate significant values ($p < 0.05$)
