# Supplementary material for: Low Self-Esteem and Life Satisfaction as a Significant Risk Factor for Eating Disorders among Adolescents
Source: Nutrients. 2023 Mar 25;15(7):1603. doi: 10.3390/nu15071603 (PMC10096620; doi:10.3390/nu15071603)
Supplement: Supplementary file 1 [file nutrients-15-01603-s001.zip › Supplementary Table S3.pdf]

Supplementary Table S3. Treatment for eating disorders and self-esteem.

Treatment for eating disorders	Basic descriptive statistics							
	n	Medium	Median	Min.	Max.	Quartile I	Quartile III	Standard deviation
**Yes**	20	25,90	26,50	19,00	33,00	22,50	28,00	3,65
**No**	213	23,68	24,00	13,00	33,00	21,00	26,00	3,86
**Total**	233	23,87	24,00	13,00	33,00	22,00	27,00	3,89
	Z=2,26 p=0,023							

Z- Mann-Whitney U test result; red color indicate significant values ($p < 0.05$)
